# In vivo evidence for cell body loss in cortical lesions in people with multiple sclerosis

**DOI:** 10.1002/acn3.52237

**Published:** 2024-12-13

**Authors:** Eva A. Krijnen, Hansol Lee, Samantha Noteboom, Florence L. Chiang, Martijn D. Steenwijk, Menno M. Schoonheim, Eric C. Klawiter, Susie Y. Huang

**Affiliations:** ^1^ Department of Neurology, Massachusetts General Hospital Harvard Medical School Boston Massachusetts 02114 USA; ^2^ MS Center Amsterdam, Anatomy and Neurosciences, Vrije Universiteit Amsterdam, Amsterdam Neuroscience Amsterdam UMC location VUmc 1007 MB Amsterdam The Netherlands; ^3^ Athinoula A. Martinos Center for Biomedical Imaging, Department of Radiology, Massachusetts General Hospital Harvard Medical School Charlestown Massachusetts 02129 USA

## Abstract

**Objective:**

To quantify alterations in soma and neurite density imaging measures within and surrounding cortical lesions in people with multiple sclerosis using in vivo high‐gradient diffusion MRI.

**Methods:**

In this cross‐sectional study, 41 people with multiple sclerosis and 34 age‐ and sex‐matched healthy controls underwent 3 T high‐gradient diffusion MRI. Cortical lesions were segmented on artificial intelligence‐enabled double inversion recovery images. “Inner” and “outer” perilesional layers were segmented as two expanding shells of 2 mm surrounding a cortical lesion. Intracellular, intra‐neurite, and extracellular signal fractions and apparent soma radius were estimated in (peri)lesional and normal‐appearing cortex.

**Results:**

Cortical lesions were present in all people with multiple sclerosis with a median count of 8 [IQR 5–18] and total volume of 0.16 [0.09–0.46 mL]. People with multiple sclerosis (mean 0.27 ± 0.03) showed lower normalized cortical volumes compared to healthy controls (0.30 ± 0.02). Compared to healthy controls (mean 0.58 ± 0.028), normal‐appearing cortex in multiple sclerosis (0.57 ± 0.034) showed lower intra‐cellular signal fraction. Cortical lesions (0.49 ± 0.089) exhibited lower intra‐cellular signal fractions compared to perilesional (“inner”: 0.55 ± 0.049, “outer”: 0.55 ± 0.039) and normal‐appearing cortex, demonstrating a gradation of change. The soma radius varied significantly across cortices, becoming smaller when moving outward from cortical lesions (cortical lesions: 10.38 ± 0.209 μm, “inner” layer: 10.19 ± 0.140 μm, “outer” layer: 10.07 ± 0.149 μm, normal‐appearing cortex: 9.99 ± 0.127 μm).

**Interpretation:**

Cortical cell body loss in multiple sclerosis is most pronounced in cortical lesions and also present in normal‐appearing cortex. Gradients of diffusion microstructural alterations moving outward from cortical lesions toward normal‐appearing cortex highlight the potential of high‐gradient diffusion MRI to identify both focal and diffuse cortical pathology.

## Background

Multiple sclerosis (MS) is an inflammatory, demyelinating, and neurodegenerative disease of the central nervous system. In addition to demyelination and axonal damage in the white matter (WM), an array of pathology affects the gray matter (GM) in MS, including demyelinating lesions, meningeal inflammation, neuronal injury, and Wallerian or trans‐synaptic degeneration, ultimately resulting in widespread atrophy.^1^ However, quantifying the precise cellular and axonal processes in the MS cortex and the subsequent clinical progression remains difficult, limiting the assessment of therapies to halt or slow down disease progression.

Cortical neuroaxonal degeneration accompanies neuroinflammation at disease onset,^2^ predominating in regions of overt demyelination, that is, cortical lesions (CLs), but also extending to the normal‐appearing tissue.^3^, ^4^ CLs are associated with irreversible neurodegeneration and cognitive impairment in people with MS.^5^, ^6^ Imaging measures of cortical tissue damage, including the diffusion tensor imaging metric mean diffusivity, can help reveal the structural impact on cognitive impairment.^7^ However, the pathophysiology underlying cortical neurodegeneration, including the potential contributions of focal cortical demyelination, remains unclear.

High‐gradient diffusion MRI is sensitive to cellular and axonal loss in MS and offers a noninvasive means of probing the microstructural substrate of neurodegeneration in GM and WM with greater specificity than conventional diffusion models like diffusion tensor imaging.^8^, ^9^, ^10^ Recently introduced biophysical compartment models such as the soma and neurite density imaging (SANDI) model have been applied to high‐gradient diffusion MRI data to reveal widespread reductions in cortical and deep GM cell body density in MS.^9^ Additionally, declines in cortical cell body density were significantly associated with thalamic volume loss,^9^ possibly mediated by neuroaxonal damage in the intervening WM.^11^ Interestingly, cortical cell body density was not significantly associated with cortical volume loss in a cohort with relatively mild disability.^9^ Heterogeneous processes such as focal inflammation and demyelination in the cortex may modulate this relationship, making it intriguing to further identify their microstructural properties in vivo.

The goal of this work was to quantify alterations in cell body and neurite densities within and surrounding CLs in MS using high‐gradient diffusion MRI. We hypothesized that cortical cell body density would be reduced within CLs and demonstrate a perilesional gradient of decreasing cell body density from non‐lesional to lesional cortex in MS. We systematically assessed regional alterations in cortical tissue microstructure as quantified by SANDI metrics within and surrounding CLs identified by a novel artificial intelligence‐enabled double inversion recovery (aiDIR) contrast.

## Methods

### Participants

This cross‐sectional study was approved by the Massachusetts General Hospital Institutional Review Board. Data from this study have been used in previous publications studying high‐gradient diffusion MRI measures, but tissue microstructure in CLs has not been investigated before.^8^, ^9^, ^11^, ^12^ All participants provided written informed consent. Participants with MS had to meet McDonald diagnostic criteria for clinically definite MS^13^ without relapses for at least 3 months, either receiving stable disease‐modifying treatment or no treatment for at least 6 months. Participants with other neurological or psychiatric brain diseases and contraindications to MRI were excluded. Of the initial dataset, 41 people with MS and 34 age‐ and sex‐matched healthy controls (HC) were included.

### Clinical and cognitive assessments

A board‐certified neurologist blinded to imaging conducted a standard clinical examination including the Expanded Disability Status Scale as a broad measure of clinical disability. All people with MS underwent neuropsychological testing based on the MACFIMS battery.^14^ Individual test scores were corrected for the effects of age and level of education if applicable, and transformed into *Z*‐scores based on normative data. *Z*‐scores of individual tests were classified into six predefined cognitive domains: verbal memory (reflected by the total learning and delayed free recall of the California Verbal Learning Test II), visuospatial memory (total and delayed free recall of the Brief Visuospatial Memory Test‐Revised), verbal fluency (total score of the Controlled Oral Word Association Test), visuospatial processing (total score of the Judgment of Line Orientation), information processing speed – working memory (3″ total score of the Paced Auditory Serial Addition Test and total score of the Symbol Digit Modalities Test), and executive functioning (total confirmed correct sorts and description score of the Delis–Kaplan Executive Function System Free Sorting Test). An average cognition *Z*‐score was calculated based on the *Z*‐scores of individual domains. People with MS were considered impaired on a cognitive domain if the domain *Z*‐score was ≤ −1.5SD below normative data.

### Image acquisition

All participants underwent brain imaging on a 3 Tesla MRI scanner (MAGNETOM Connectom; Siemens Healthineers, Erlangen, Germany) equipped with a maximum gradient strength of 300 mT/m and a custom‐built 64‐channel head coil.^15^ Diffusion data were acquired using a diffusion‐weighted spin‐echo single‐shot EPI sequence (echo time/repetition time 77/3600 ms, simultaneous multi‐slice imaging with slice acceleration factor 2, parallel imaging acceleration factor *R* = 2, anterior‐to‐posterior phase encoding, 2 mm^3^ isotropic resolution, sagittal slices, acquisition time 51 min), following a previously established protocol.^8^, ^16^ Non‐diffusion‐weighted images (*b* = 0) were acquired every 16 images. For fitting of the SANDI model, diffusion data acquired at diffusion times of Δ = 19 at eight *b*‐values (*b* = 50–350–800–1500 s/mm^2^ in 32 directions, and *b* = 2400–3450–4750–6000 s/mm^2^ in 64 directions) were used. Distortions due to susceptibility effects were corrected by means of five *b* = 0 images with a reversed‐phase encoding direction.

Additionally, structural brain data were acquired with the use of a T_1_‐weighted multi‐echo magnetization prepared rapid acquisition gradient echo (MEMPAGE) sequence (echo time/repetition time/inversion time 1.15–3.03–4.89–6.75/2530/1100 ms, *R* = 3, flip angle 7°, 1 mm^3^ isotropic resolution, acquisition time 3 min 58 sec) and 3D fluid‐attenuated inversion recovery (FLAIR) sequence (echo time/repetition time/inversion time 389/5000/1800 ms, *R* = 2, 0.9 mm^3^ isotropic resolution, acquisition time 5 min 47 sec).

### Data processing

#### White matter lesion segmentation

Lesion segmentation of FLAIR hyperintensities was performed using a validated FreeSurfer‐based automated segmentation tool.^17^ A board‐ and subspecialty‐certified neuroradiologist manually edited the lesion masks (S.Y.H.).

#### Cortical lesion detection and segmentation

**Figure 1:**
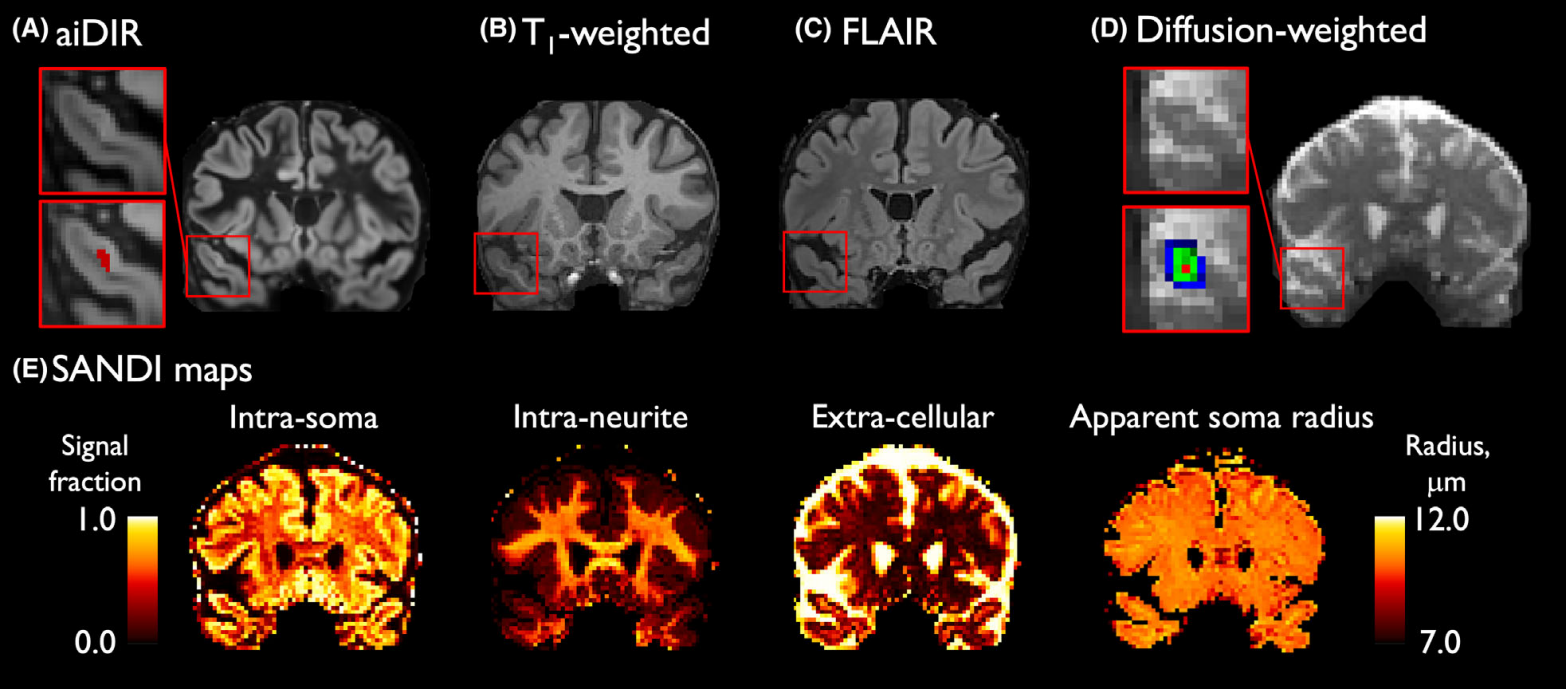
Representative coronal images of the various contrasts used in the cortical lesion segmentation pipeline. Cortical lesions (in red boxes) were delineated on artificial intelligence‐enabled double inversion recovery contrast images (aiDIR; A) with the 3D T_1_‐weighted (B) and T_2_‐weighted fluid‐attenuated inversion recovery contrast (FLAIR; C) images as inputs. In diffusion space (D), the red voxels correspond to the lesion core, green to the inner perilesional layer, and blue to outer perilesional layer. Maps of individual soma and neurite density imaging measures (SANDI; E) are shown with signal fractions ranging from 0 to 1, and the soma radius from 7 to 12 μm.

For each participant, an aiDIR image for CL detection was generated by a fully convolutional neural network with the use of the T_1_‐weighted and FLAIR images as inputs, which has recently been validated in a multicenter study (Fig. 1A–C).^18^, ^19^ Following MAGNIMS consensus guidelines,^20^ CLs were scored and segmented using Slicer (version 4.6.2) on the aiDIR images by an experienced reader (E.A.K.) supervised by an experienced board‐certified neuroradiologist (S.Y.H.). Both readers were blinded to the participant characteristics during the segmentation process. This yielded a CL mask for each MS participant for assessment of lesion volume and spatial distribution.

#### Gray matter segmentation

Cortical surface and volumetric reconstruction were performed on the T_1_‐weighted images by means of FreeSurfer (version 5.3). An experienced user manually reviewed these reconstructions and filled in WM lesions disrupting the cortical boundary to prevent misclassification of GM.^21^ We obtained segmented tissue masks of WM, cortical and deep GM, and cerebrospinal fluid using the *5ttgen hsvs* function in MRtrix based on the surface reconstruction of FreeSurfer.^22^, ^23^ The segmented tissue masks were multiplied by volume fractions maps computed by FreeSurfer to derive partial volume‐weighted tissue masks for correction of partial volume averaging effects of the diffusion‐weighted volumes.

For visualization of the spatial distribution of CLs, we projected the individual CL masks on each MS participant's cortical surface generated by FreeSurfer resulting in 2D lesion surfaces with a maximum projection distance of 4 mm outward of the GM/WM border. The CL surfaces were then resampled to a common FreeSurfer surface template using the *mri_surf2surf* function, smoothed with a 10 mm full width at half maximum of Gaussian kernel.

For evaluating the diffusion microstructural MR measures within our cortical regions of interest, affine ANTs transformation parameters were applied to the CL and partial volume‐weighted tissue masks with the use of nearest‐neighbor and linear interpolation, respectively, to transform the masks to the diffusion‐weighted image.^24^ Rigid body transformation failed to align the diffusion data to the FreeSurfer T_1_‐weigted image completely due to minor residual distortions in the diffusion MRI data after preprocessing (see below). Therefore, affine transformation was applied to ensure more precise registration. Perilesional tissue was then segmented as two expanding 3D shells each measuring 2 mm in thickness surrounding every CL (“inner” and “outer” perilesional layers; Fig. 1D). Perilesional layers were masked by the partial‐volume weighted GM tissue mask to exclude cerebrospinal fluid and WM contamination. Normal‐appearing cortex was segmented by subtracting lesional and perilesional tissue masks from entire cortex maps.

Cortical volumes were calculated and normalized by the total intracranial volume provided by FreeSurfer.

#### SANDI measure calculations

Diffusion data were preprocessed using an established pipeline.^16^ Briefly, diffusion‐weighted images were corrected for gradient nonlinearity with the use of in‐house software,^25^ and any distortions due to susceptibility and eddy current effects were corrected with the use of topup and eddy in the FMRIB Software Library (version 5.0).^26^, ^27^, ^28^

For fitting of the SANDI model, we used the AMICO software^29^, applying the default diffusivity parameters and a λ2 regularization term of 0.005, as previously described.^9^ Estimates of the intra‐neurite (*f*_in_), extracellular (*f*_ec_), and intra‐soma (*f*_is_) signal fractions and apparent soma radius (*R*_s_) were obtained in CLs, perilesional and normal‐appearing cortex (Fig. 1E). To minimize potential confounding by partial volume averaging effects, we calculated partial volume‐weighted mean values per SANDI measure with the use of the partial volume‐weighted cortical GM mask.

To test the partial volume averaging effects on the small lesional regions of interest, the resolution of the diffusion data was increased to 1 mm isotropic by employing a novel model‐free “SUper‐REsolution” pipeline using self‐similarity.^30^, ^31^ The generated high‐resolution diffusion data were upsampled to the same resolution as the 3D T1‐weighted anatomical volumes, that is, 1 mm^3^ isotropic. With the use of the super‐resolved 1 mm isotropic resolution data, we again calculated partial volume‐weighted mean values per SANDI measure in CLs, perilesional and normal‐appearing cortex. These mean values were compared with the original diffusion data, yielding no significant differences between the original data and super‐resolved data in the small cortical regions of interest assessed in this study (Table S1), and without significant differences in the relative trends reported between regions of interest (see Results below).

### Statistical analysis

R (v4.2.1) was used for data analysis. Shapiro–Wilk testing and histogram inspection of the variable distribution were used to assess normality. We analyzed baseline characteristics with descriptive statistics, expressed as N (%), mean ± SD or median [IQR], depending on normality and type. Due to a non‐normal distribution, CL volume was log‐transformed for subsequent analysis.

To confirm the previously identified SANDI alterations in MS cortex versus the cortex of the included HC,^9^ comparisons between people with MS and HC were performed by independent samples *t*‐tests. We then assessed the presence of perilesional gradients of microstructural SANDI alterations moving outward from CL to normal‐appearing cortex. We applied repeated measures ANOVA for within‐subject comparisons between lesional, inner, and outer peri‐lesional layers and normal‐appearing cortex, and paired *t*‐tests for post hoc analyses between cortices. Finally, given the relationships between CL and cortical volume loss, we assessed the associations between the underlying cortical microstructural alterations observed in normal‐appearing cortex and CLs and cortical volume loss. Multivariable linear regression models explaining cortical volume loss by cortical microstructure were used to explore these relationships, adjusting for age and sex. To assess the clinical relevance for cognitive functioning of the detected cortical microstructural alterations, similar regression models were performed for the correlation between relevant SANDI measures and average cognition *Z*‐score.

The performed analyses were corrected for multiple comparisons using the false discovery rate^32^ per analysis step, that is, group comparisons between MS and HC, within‐subject comparisons for CL analyses and the regression models for the relationships between microstructure and volume loss and cognition, with *α* = 0.05, reported as *p*_corr_.

## Results

### Demographics

People with MS were of similar age and sex compared to HC (Table 1). People with MS had a mean disease duration of 9.7 ± 6.9 years, had predominantly relapsing–remitting MS (80.5%), and had mild clinical disability (median EDSS 2.5 [IQR 2.0–3.5]). Normalized cortical and deep GM volumes were significantly lower in MS compared to HC (*p* < 0.001 and *p* = 0.001, respectively).

**Table 1:** Baseline demographics and clinical characteristics of included participants.

	HC (*N* = 34)	People with MS (*N* = 41)	HC versus MS^a^
			*p*
Demographics			
Age, years	39.1 (14.8)	45.2 (12.9)	0.058
Sex, male/female [number (%)]	14 (41.0)/20 (59.0)	11 (26.8)/30 (73.2)	0.286
Education, years	–	16.2 (2.5)	–
Disease characteristics			
Disease duration, years	–	9.7 (6.9)	–
MS subtype, RR/SP/PP	–	33 (80.5)/5 (12.2)/3 (7.3)	–
DMT use^b^, yes [number (%)]	–	35 (85.4)	–
Clinical characteristics			
EDSS, score	–	2.5 [2.0–3.5]	–
Executive functioning, *Z*‐score	–	0.53 (0.97)	–
Information processing speed, *Z*‐score	–	−0.26 (1.10)	–
Verbal fluency, *Z*‐score	–	−0.10 (0.96)	–
Verbal memory, *Z*‐score	–	−0.26 (1.30)	–
Visuospatial memory, *Z*‐score	–	−0.15 (1.50)	–
Visuospatial processing, *Z*‐score	–	0.23 (0.98)	–
MR characteristics			
WM lesion load, mL	–	4.13 [1.87–10.80]	–
Cortical GM volume, mL	479.12 (58.98)	419.77 (72.79)	**2.11 × 10** ^**−4**^
Normalized cortical GM volume	0.30 (0.02)	0.27 (0.03)	**4.17 × 10** ^**−9**^
Deep GM volume, mL	44.06 (3.91)	41.85 (5.03)	**0.035**
Normalized deep GM volume	0.028 (0.002)	0.027 (0.002)	**0.001**

Variables are reported as mean (SD) or median [interquartile range] unless otherwise indicated. Significant *p*‐values (<0.05) are marked in bold.

DMT, disease‐modifying treatment; EDSS, Expanded Disability Status Scale; GM, gray matter.; MR, magnetic resonance; MS, multiple sclerosis; PP, primary progressive; RR, relapsing–remitting; SP, secondary progressive; WM, white matter.

^a^ Independent samples *t*‐test for continuous variables; chi‐square test for categorical variables.

^b^ 35 of 41 people with MS were taking MS disease‐modifying therapy (9 dimethyl fumarate, 8 glatiramer acetate, 5 fingolimod, 5 ocrelizumab, 3 interferon beta (1a/1b), 2 natalizumab, 2 rituximab, and 1 teriflunomide).

### Cortical microstructure

Considering the entire cortex, people with MS showed lower *f*_is_ and a trend toward higher *f*_ec_ compared to HC (*p*_corr_ = 0.030 and *p*_corr_ = 0.085; Table 2). After adjusting for age and sex, only *Z*‐scores relative to HC of mean cortical *R*_s_ were significantly associated with *Z*‐scores of normalized cortical volumes in people with MS [*Β*(95% confidence interval, CI) = 0.31 (0.11; 0.50), *p*_corr_ = 0.010]. Cortical *f*_is_, *f*_in_, and *f*_ec_ were not significantly associated with normalized cortical volume [*f*_is_: *Β*(95% CI) = 0.06 (−0.30; 0.41), *p*_corr_ = 0.746; *f*_in_: *Β*(95% CI) = 0.06 (−0.32; 0.45), *p*_corr_ = 0.746; and *f*_ec_: *Β*(95% CI) = −0.05 (−0.31; 0.22), *p*_corr_ = 0.746].

**Table 2:** Group differences in cortical soma and neurite density imaging measures.

	HC (*N* = 34)	MS (*N* = 41)	Mean difference (95% CI)	Effect size	*p*	*p*_corr_
Entire cortex						
*f*_is_	0.58 (0.028)	0.56 (0.033)	−0.019 (0.028)	0.62	**0.007**	**0.030**
*f*_in_	0.17 (0.015)	0.17 (0.016)	0.005 (0.014)	−0.31	0.173	0.173
*f*_ec_	0.25 (0.024)	0.26 (0.036)	0.014 (0.028)	−0.46	**0.042**	0.085
*R*_s_	10.02 (0.069)	9.99 (0.12)	−0.032 (0.091)	0.31	0.158	0.173

Intra‐soma (*f*_is_), intra‐neurite (*f*_in_), and extracellular (*f*_ec_) signal fractions and apparent soma radius (*R*_s_ in μm) in the entire cortex were compared between people with multiple sclerosis (MS) and healthy controls (HC) using independent samples *t*‐tests. Mean differences with 95% confidence intervals (CI) and effect sizes (Hedges' *g*) are reported with corresponding *p*‐values. *p*‐values were corrected for multiple comparisons with the use of false discovery rate, reported as *p*_corr_. Significant *p*‐values (<0.05) are marked in bold.

### Cortical lesion distribution

**Figure 2:**
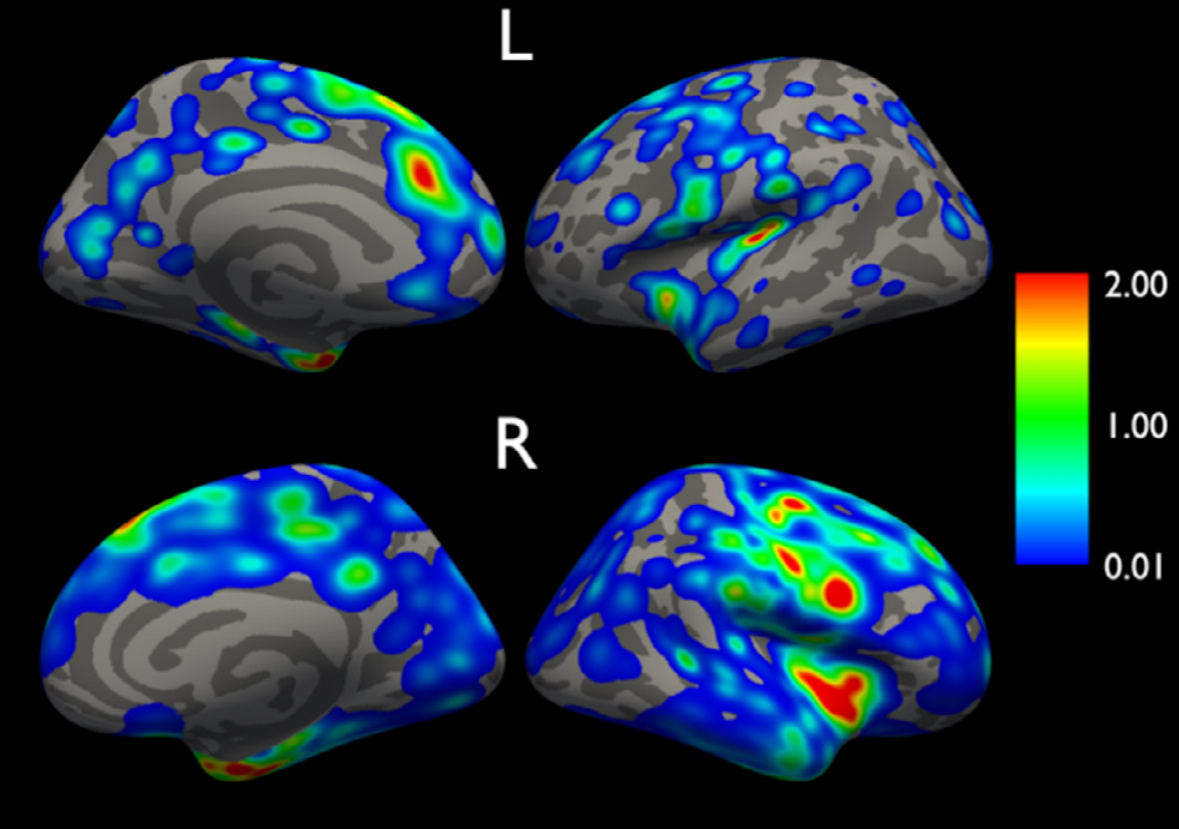
Cortical lesion distribution map. Smoothed (10 mm) cortical lesion surfaces of all individuals ranged from 0 (no lesion) to 1 (lesion core). Surfaces were summed across people with multiple sclerosis, yielding cortical lesions probability maps displaying at a range of 0–2.

All people with MS had at least one CL, with a median count of 8 [IQR 5–18] and total volume of 0.16 mL [IQR 0.09–0.46 mL]. Locations with the highest probabilities of identifying CLs were the left and right insular cortex, temporal poles, entorhinal areas, and superior frontal and precentral gyri (Fig. 2).

In MS, the log‐transformed total CL volume was significantly associated with normalized cortical volume *Z*‐scores relative to HC [*Β*(95% CI) = −0.34 (−0.67;‐0.01), *p* = 0.043]. With regard to cortical microstructure, *Z*‐scores relative to HC of mean cortical *f*_in_ were significantly associated with log‐transformed total CL volume [*Β*(95% CI) = −0.31 (−0.59;‐0.03), *p* = 0.030]. A trend toward a significant association between cortical *f*_ec_ and log‐transformed total CL volume [*Β*(95% CI) = 0.36 (−0.06; 0.77), *p* = 0.091] was observed, whereas cortical *f*_is_ and *R*_s_ were not associated with log‐transformed total CL volume [*Β*(95% CI) = −0.14 (−0.47; 0.18), *p* = 0.377; and *Β*(95% CI) = −0.17 (−0.69; 0.36), *p* = 0.531, respectively]. None of the significant associations survived false discovery rate‐correction.

### Cortical lesion microstructure

CLs showed lower *f*_is_ (*p*_corr_ <0.001), higher *f*_in_ and *f*_ec_ (*p*_corr_ = 0.017 and *p*_corr_ <0.001, respectively) and larger *R*_s_ (*p*_corr_ <0.001) compared to HC cortex (Table 3). The inner perilesional layer showed significantly lower *f*_is_, higher *f*_in_, and larger *R*_s_ (all *p*_corr_ <0.001) compared to HC cortex. The outer perilesional layer showed lower *f*_is_ (*p*_corr_ <0.001) and higher *f*_in_ and *f*_ec_ (*p*_corr_ = 0.003 and *p*_corr_ = 0.017, respectively) compared to HC cortex. Normal‐appearing cortex in MS showed significantly lower *f*_is_ compared to HC cortex (*p*_corr_ = 0.014).

**Table 3:** Regional alterations in cortical microstructural measures in people with multiple sclerosis.

	HC (*N* = 34)	MS (*N* = 41)	Mean difference (95% CI)	Effect size	*p*	*p*_corr_
Normal‐appearing cortex						
*f*_is_	0.58 (0.028)	0.57 (0.034)	−0.019 (0.028)	0.62	**0.008**	**0.014**
*f*_in_	0.17 (0.015)	0.17 (0.015)	0.005 (0.013)	−0.35	0.130	0.138
*f*_ec_	0.25 (0.024)	0.26 (0.036)	0.014 (0.028)	−0.44	**0.049**	0.061
*R*_s_	10.02 (0.069)	9.99 (0.127)	−0.033 (0.092)	0.32	0.152	0.152
Outer perilesional layer						
*f*_is_	–	0.55 (0.039)	−0.035 (0.030)	1.04	**1.57 × 10** ^**−5**^	**5.02 × 10** ^**−5**^
*f*_in_	–	0.18 (0.030)	0.018 (0.021)	−0.73	**0.001**	**0.003**
*f*_ec_	–	0.27 (0.035)	0.018 (0.028)	−0.59	**0.011**	**0.017**
*R*_s_	–	10.07 (0.149)	0.055 (0.106)	−0.46	**0.040**	0.054
Inner perilesional layer						
*f*_is_	–	0.55 (0.049)	−0.036 (0.036)	0.89	**1.60 × 10** ^**−4**^	**3.65 × 10** ^**−4**^
*f*_in_	–	0.19 (0.030)	0.021 (0.021)	−0.90	**1.34 × 10** ^**−4**^	**3.56 × 10** ^**−4**^
*f*_ec_	–	0.27 (0.042)	0.015 (0.032)	−0.43	0.056	0.065
*R*_s_	–	10.19 (0.140)	0.174 (0.101)	−1.52	**3.52 × 10** ^**−9**^	**2.82 × 10** ^**−8**^
Cortical lesions						
*f*_is_	–	0.49 (0.089)	−0.091 (0.059)	1.32	**1.71 × 10** ^**−7**^	**9.09 × 10** ^**−7**^
*f*_in_	–	0.18 (0.041)	0.018 (0.027)	−0.57	**0.012**	**0.017**
*f*_ec_	–	0.32 (0.086)	0.074 (0.057)	−1.12	**4.67 × 10** ^**−6**^	**1.87 × 10** ^**−5**^
*R*_s_	–	10.38 (0.209)	0.3 58 (0.141)	−2.20	**1.14 × 10** ^**−13**^	**1.83 × 10** ^**−12**^

Intra‐soma (*f*_is_), intra‐neurite (*f*_in_), and extracellular (*f*_ec_) signal fractions and apparent soma radius (*R*_s_ in μm) were assessed in the normal‐appearing cortex, the outer and inner perilesional layers, and cortical lesions relative to healthy cortex with the use of independent samples *t*‐tests. Mean differences with 95% confidence intervals (CI) and effect sizes (Hedges' *g*) are reported with corresponding *p*‐values. *p*‐values were corrected for multiple comparisons with the use of false discovery rate, reported as *p*_corr_. Significant *p*‐values (<0.05) are marked in bold.

**Figure 3:**
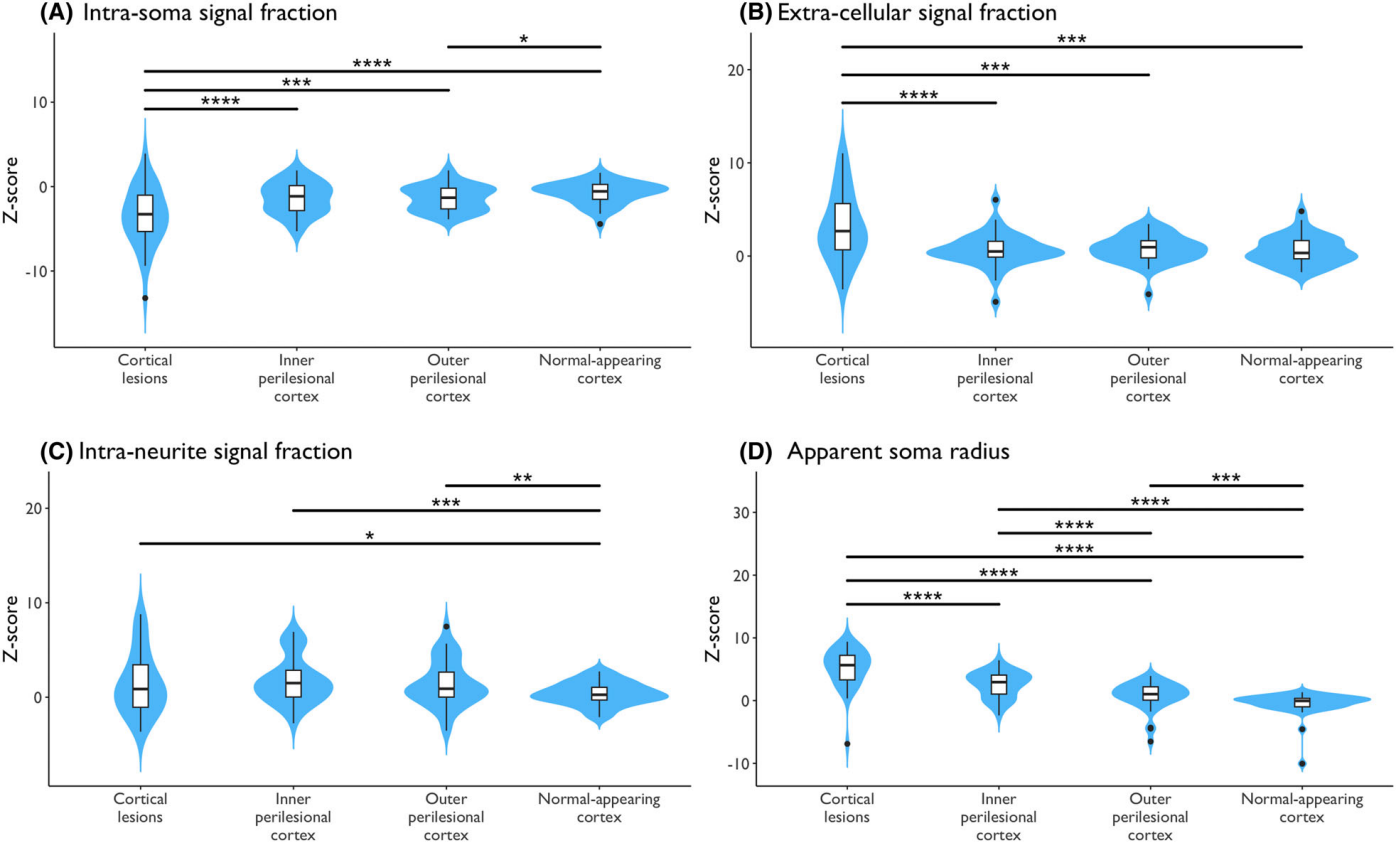
Microstructural measures in cortical lesions, perilesional layers, and normal‐appearing cortex in people with multiple sclerosis. Each boxplot displays the distribution of values for the specified cortical region. The box shows the median value (thick black centre line), extending from the 25th to the 75th percentile of the distribution, while the vertical lines indicate the range of adjacent values (1.5 times the interquartile range from the quartiles); dots denote observations outside this range. All significant within‐subject differences survived correction for multiple comparisons with the use of false discovery rate. Asterisks correspond to the level of significance before correction: * = <0.05, ** = <0.01, *** = <0.001, and **** = <0.0001.

Within‐subject alterations in SANDI measures are displayed in Figure 3. CLs exhibited lower *f*_is_ and higher f_ec_ compared to perilesional and normal‐appearing cortex (*p*_corr_ <0.001 for all). Furthermore, the outer perilesional layer showed lower *f*_is_ than normal‐appearing cortex (*p*_corr_ = 0.045). Both perilesional layers showed higher *f*_in_ compared to normal‐appearing cortex (inner: *p*_corr_ <0.001 and outer: *p*_corr_ = 0.005). *R*_s_ varied significantly across all cortical regions of interest, becoming smaller and approaching values comparable with HC cortex when moving outward from CLs to normal‐appearing cortex (all *p*_corr_ <0.001). Of note, the relative within‐subject differences in SANDI measures between cortical regions of interest remained consistent between the original and the super‐resolved 1 mm isotropic resolution data, with lower intra‐soma and higher extracellular signal fractions, and larger soma radius observed when moving from normal‐appearing cortex to CLs. In the sub‐sample of eight individuals with MS in whom we were able to obtain super‐resolution data, the effect sizes for the signal fractions were larger (*f*_is_: *F* (1.4, 10.0) = 29.5, *p* < 0.001; *f*_in_: *F* (1.4, 9.5) = 2.5, *p* = 0.140; *f*_ec_: *F* (3, 21) = 28.7, *p* < 0.001), whereas the effect size of the soma radius (*R*_s_: *F* (3, 21) = 39.9, *p* < 0.001) appeared smaller in the upsampled data compared to the original diffusion data (*f*_is_: *F* (1.4, 10.0) = 16.9, *p* = 0.001; *f*_in_: *F* (3, 21) = 0.4, *p* = 0.749; *f*_ec_: *F* (1.2, 8.3) = 11.1, *p* = 0.008; *R*_s_: *F* (3, 21) = 58.8, *p* < 0.001).

### Cortical damage and cognitive function

Our cohort of predominantly relapsing–remitting MS had a mean average cognition *Z*‐score of 0, reflecting none to mild overall cognitive impairment in our sample. Lower *Z*‐scores of normalized cortical volumes relative to HC tended to be associated with lower average cognition *Z*‐scores [*Β*(95% CI) = 0.16 (−0.03; 0.35), *p* = 0.096; Fig. S1A]. With regard to cortical microstructural measures, regression models showed a trend toward a positive relationship between lower cortical *f*_is_, particularly of normal‐appearing cortex, and worse cognition [*Β*(95% CI) = 0.17 (−0.03; 0.37), *p* = 0.098; Fig. S1B]. Fifteen (37%) people with MS were impaired on ≥1 cognitive domain and showed trends toward lower *f*_is_ *Z*‐scores in normal‐appearing cortex (−1.25 ± 1.32; *p* = 0.063) and higher *f*_is_ *Z*‐scores in CLs (−2.18 ± 3.23; *p* = 0.056) compared to non‐impaired participants (−0.45 ± 1.22 and − 4.31 ± 3.39, respectively). Other SANDI measures and CL volume did not show significant associations with cognition (Table S2).

## Discussion

The goal of this study was to quantify alterations in cell body and neurite densities within and surrounding CLs in MS using a high‐gradient diffusion model optimized for the cortex and a novel aiDIR contrast for identifying CLs. Our group previously characterized alterations in SANDI measures in the cortex in MS but did not evaluate these changes with respect to CLs.^9^ In the current study, we showed that cortical *f*_is_ was reduced in MS lesions, suggestive of cell body loss, and showed a perilesional gradient of decreasing cell body density from non‐lesional to lesional cortex. Similar gradients distinguishing lesional and perilesional tissue were seen with higher *f*_ec_ and *R*_s_ in lesions, reflecting expansion of the extracellular space and larger cell size within CLs. Lower cortical volumes were significantly associated with smaller cortical cell size but not with cortical cell body or neurite densities. Regarding the extent of focal cortical demyelination, higher CL volume was associated with lower cortical volume and lower cortical *f*_in_. The *f*_in_ was higher in perilesional and lesional cortex compared to HC cortex. Cognitive function showed a potential relationship with cortical volume and cortical cell body density.

### Cortical lesion microstructure

The prevalence of CLs was highest in the insular, temporal, and frontal regions. This finding is in line with previous 7 T MRI research showing a predominance of leukocortical lesions in these regions.^33^ Of note, leukocortical lesions are the CL subtype most commonly described on 3 T DIR, as subpial demyelination often goes undetected.^18^ Compared to the normal‐appearing cortex, alterations in *f*_is_, *f*_ec_, and *R*_s_ were gradually more pronounced moving toward the lesional cortex. Subpial demyelination exhibits neuronal densities akin to those observed in normal‐appearing cortex,^34^ supporting our contention that the leukocortical lesions evaluated in this study are primarily responsible for the overall cortical microstructure changes. Previous studies utilizing lower gradient strength diffusion MRI also detected lower cell body densities in the MS cortex compared to healthy cortex,^35^, ^36^ with further localized decreases in leukocortical lesions compared to subpial lesions and subpial lesions displaying values closer to the surrounding normal‐appearing cortex.^35^ Cell body density values in CLs showed greater variability than in normal‐appearing cortex. This variability may reflect the heterogeneity of focal cortical pathology in terms of inflammatory, neurodegenerative and remyelinating activity observed in histopathology and PET‐MRI studies. For example, cortex is characterized by reduced neuronal density, potentially caused by the loss of trophic support from oligodendrocytes, compared to normal‐appearing and HC cortex.^2^, ^37^, ^38^ Increased inflammatory activity is also seen, including myelin/lipid‐laden macrophages, lymphocytic infiltrates, and reactive oligodendrocytes and microglia, indicating ongoing demyelination.^2^, ^37^, ^38^ This increased inflammatory activity is predominantly present in leukocortical lesions, that is, lesions most prominent at our 3 T field strength, compared to intracortical and subpial lesions.^39^, ^40^ Whether the SANDI alterations observed in our study are a result of these underlying processes should be addressed in future imaging‐histopathological correlation studies.

Two hypotheses may explain the increased *R*_s_ and diminished *f*_is_ observed in CLs, which are both tied to the shape and function of glial cells within the cortex. First, the observed reduction in cell body signal fraction may reflect the apoptosis of astrocytes and oligodendrocytes,^2^, ^41^ which, in their *non‐reactive* state, are smaller than neuronal cell bodies and are about a one‐quarter fewer in number compared to neurons.^42^ Alternatively, it may reflect significant neuronal apoptosis^2^, ^37^, ^41^ alongside *reactive* astrocytes, which appear larger in size compared to their *non‐reactive* state, suggesting either persistent inflammation or remyelination.^41^ Previous histopathology work in the MS spinal cord showed an association between higher *f*_is_ and glial fibrillary acidic protein (GFAP) staining, reflecting astrocytes, in lesional WM.^43^ Future imaging‐histopathological correlation studies in the MS brain should assess whether the loss of smaller cells results in a decrease in *f*_is_ while the presence of activated glia may result in a relative increase in overall cell body radius in lesional cortex. A trend toward larger *R*_s_ in lesional cortex has been described in preliminary work applying the SANDI model in a small sample of individuals with MS.^44^ Future postmortem validation of these specific findings is therefore a logical next step.

Based on histopathology, lower *f*_in_ appears associated with lower myelin intensities (myelin proteolipid protein staining) within the lesional WM.^43^ The observed *increase* in *f*_in_ specifically in (peri)lesional cortex compared to HC cortex may be further validated through specific microglia staining in lesional and perilesional tissue combined with a regional assessment of the SANDI measures. For example, these changes may be attributed to alterations in the shape and density of reactive microglia and their associated cellular processes. Reactive microglia can appear at the border of lesional cortex and are characterized by larger cell bodies and thicker cell processes.^45^

### Relationships between cortical volume, microstructure, and lesions

In our cohort, cortical volume was positively associated with cell body size. Based on an experimental autoimmune encephalomyelitis animal model, neurodegeneration may be accompanied by decreased residual neuronal size mediated by axonal or dendritic loss and subsequently inducing cortical volume loss.^41^ Conversely, CL volume showed a negative relationship with cortical volume, in line with previous literature.^5^, ^6^ We did not observe a relationship between CL volume and *f*_is_, which may be supported by histopathologic studies showing that the percentage of cortical demyelination is not proportional to cortical neuronal density.^46^ The cross‐sectional design of this study limits our ability to evaluate associations with temporal relationships.

### Cortical microstructure and cognitive function

Loss of cortical volume and *f*_is_ tended to be associated with worse cognition. Specifically, cell body density in normal‐appearing cortex was more closely associated with cognition scores than volume of lesional cortex, underscoring the importance of microstructural alterations in normal‐appearing cortex in contributing to cognitive dysfunction.^7^ The highly heterogeneous pathological processes occurring at various stages within CLs may possibly explain the absence of associations between cortical microstructure and cognitive function in this study, along with the relatively mild degree of cognitive impairment in our sample of people with MS. Trends observed between cortical volume loss, lower *f*_is_ and worse cognition emphasize the clinical potential of cortical diffusion measures for identifying people with MS at risk for future cognitive decline. Larger and longitudinal cohort studies of high‐gradient diffusion MRI in people with MS with a greater degree of cognitive impairment are needed to determine the full extent of these findings across the disease spectrum.

### Limitations

Limitations of this study include the spatial resolution of our diffusion data, which constrained our ability to probe the specific spatial variability of microstructural alterations within the cortex. Higher spatial resolution including advanced submillimeter diffusion MRI acquisitions would enable profiling of diffusion measures across cortical layers at variable depths.^47^ Partial volume effects were mitigated to the extent possible, with the caveat that newer super‐resolution methods may be applied in the future to reduce these effects further. Our exploratory analyses using super‐resolution data in a subset of people with MS revealed larger effect sizes in intra‐soma and extracellular signal fraction at higher spatial resolution, likely due to reduction in partial volume effects and in line with findings observed in other studies of super‐resolution imaging data.^48^ While we cannot exclude the effect of residual partial volume averaging on our results in the larger cohort, our findings regarding the impact of spatial resolution on the reported SANDI metrics in the cortex suggests that lower spatial resolution may decrease sensitivity to the relative differences in signal fractions observed within lesional, peri‐lesional, and normal‐appearing cortex. The fact that we were still able to detect significant differences in intra‐soma and extracellular signal fractions within lesional, peri‐lesional, and normal‐appearing cortex in MS suggests that higher spatial resolution will boost the observable effect size in applying SANDI and other advanced microstructural models to studies of cortical pathology in MS and should be investigated further in future studies. Furthermore, despite the promise of the aiDIR contrast in enhancing CL detection compared to clinical FLAIR sequences, a considerable proportion of CLs, especially subpial lesions, remain undetected and not specifically studied.^49^ It is possible those effects could relate to findings in the cortex described herein as normal‐appearing. Future endeavors may involve integrating 7 T and high‐gradient diffusion data to address this challenge. Previous studies using advanced diffusion measures have demonstrated age‐related variability in cell and neurite densities across different cortical regions.^50^, ^51^ Therefore, adopting a regional approach to assess the microstructural alterations in CLs compared to non‐lesional cortex, for example, by comparing the identified CLs with the normal‐appearing cortex in the same location in the contralateral hemisphere, would be an intriguing future direction. Our cross‐sectional study design restricts our ability to make conclusions regarding the sequence of neuroinflammatory and neurodegenerative processes following acute cortical demyelination. Ongoing longitudinal investigations seek to elucidate the temporal sequence of events. Finally, while our study highlights the advantages of high‐gradient diffusion MRI, such technologies are not yet commonly available in routine clinical settings. Conventional and other multi‐compartment diffusion MRI analytic models, such as diffusion tensor imaging and neurite orientation dispersion and density imaging, can detect differences between regions of interest with the same directionality, though to a lesser extent compared to SANDI measures,^44^, ^50^ and may be more easily accessible in clinical routine. However, SANDI may offer greater biological specificity, particularly in more complex tissues like GM, while ensuring comparable repeatability and reproducibility to those of.^44^ The introduction of high‐performance gradients in the latest generation of MRI scanners designed for clinical research should accelerate the adoption of these methods.^52^ Furthermore, efforts to apply the advanced SANDI model for inferring cortical GM microstructure using lower gradient systems show promise in offering viable alternatives.^35^, ^36^

## Conclusion

Our results provide *in vivo* evidence of microstructural alterations, including cell body loss, contributing to cortical pathology in MS, and potentially reflecting neuroinflammation and neurodegeneration occurring within and just outside CLs. Cortical cell body loss in MS is most pronounced in CLs but also present in normal‐appearing cortex. The detection of diffusion microstructural alterations in lesional and normal‐appearing cortex highlights the potential of high‐gradient diffusion MRI to identify both diffuse and focal cortical pathology. Such findings hold promise for sensitive detection of microstructural alterations within the cortex and better understanding of the impact on cognitive decline, prior to the onset of irreversible cortical volume loss and permanent disability.

## Author Contributions

E.A.K. contributed to the conception and design of the study, acquisition, and analysis of data, and drafting the manuscript and figures. H.L., S.N., and F.L.C. contributed to the acquisition and analysis of data and drafting the manuscript. M.D.S. and M.M.S. contributed to drafting the manuscript. S.Y.H. and E.C.K. contributed to the conception and design of the study, acquisition, and drafting the manuscript and figures.

## Conflict of Interest

E.A.K., H.L., and F.L.C. report no conflicts of interest; S.N. is supported by research grants from Atara Biotherapeutics, Merck and Biogen; M.D.S. is supported by research grants from Atara Biotherapeutics, Merck and Biogen; M.M.S. serves on the editorial board of Neurology and Frontiers in Neurology and Multiple Sclerosis Journal, receives research support from the Dutch MS Research Foundation, Eurostars‐EUREKA, ARSEP, Amsterdam Neuroscience, MAGNIMS, and ZonMW and has served as a consultant for or received research support from EIP Pharma, Atara Biotherapeutics, Biogen, Celgene/Bristol Meyers Squibb, Genzyme, MedDay and Merck; S.Y.H. has received consulting fees and research grants from Siemens Healthineers; E.C.K. has received consulting fees from EMD Serono, Genentech, INmune Bio, Myrobalan Therapeutics, OM1and TG Therapeutics, and received research funds from Abbvie, Biogen, and Genentech.

## Supporting information


Data S1.

## Data Availability

The tabulated data that support our findings are available from the corresponding author, upon reasonable request.
