# Supplementary material for: In vivo evidence for cell body loss in cortical lesions in people with multiple sclerosis
Source: Ann Clin Transl Neurol. 2024 Dec 13;12(1):4–16. doi: 10.1002/acn3.52237 (PMC11752088; doi:10.1002/acn3.52237)
Supplement: Supplementary file 1 — Data S1. [file ACN3-12-4-s001.docx]

**Supplementary Figure 1** **Relationships between cortical MR measures and cognitive functioning**
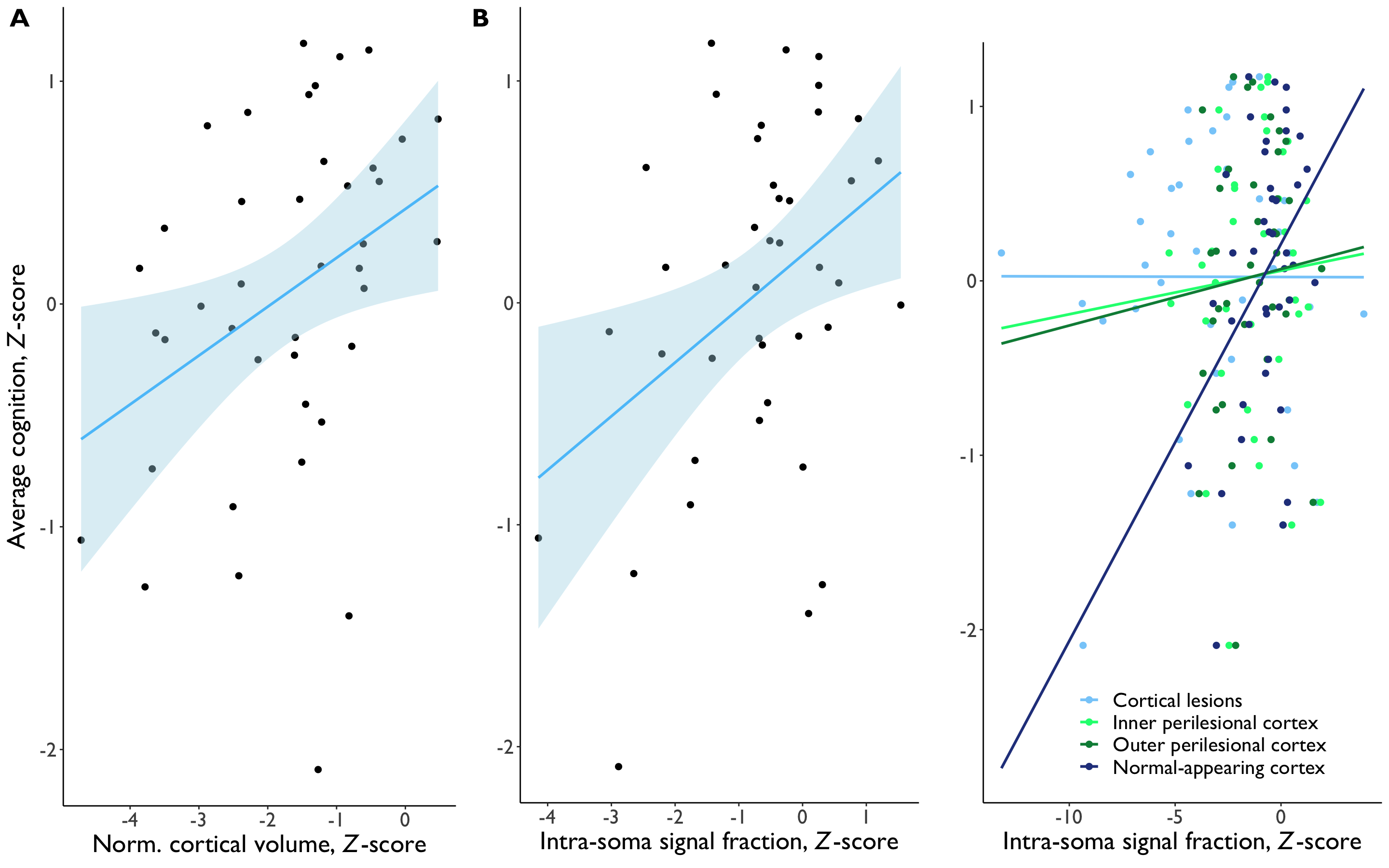


Scatter plots are shown for *Z*-scores relative to healthy controls of average cognition and normalized cortical volume (A) and cortical intra-soma signal fraction (B) in people with multiple sclerosis. In the right panel of B, average cognition Z-score is plotted against intra-soma signal fractions in cortical lesions, perilesional layers and normal-appearing cortex separately.

**Supplementary Table 1. Results of comparisons between original diffusion resolution and super-resolved 1 mm isotropic resolution data**

|  | | MS total cohort | MS subset in original diffusion resolution | MS subset in “upsampled” diffusion resolution | Original versus “upsampled” data |
| --- | --- | --- | --- | --- | --- |
|  |  | *(N=41)* | *(N=8)* | *(N=8)* | *P-value* |
| **Normal-appearing cortex** | f_is_ | 0.57 (0.034) | 0.57 (0.012) | 0.59 (0.012) | **0.007** |
|  | f_in_ | 0.17 (0.015) | 0.16 (0.010) | 0.15 (0.008) | **0.002** |
|  | f_ec_ | 0.26 (0.036) | 0.27 (0.016) | 0.27 (0.010) | 0.793 |
|  | R_s_ | 9.99 (0.127) | 10.02 (0.035) | 9.95 (0.065) | **0.023** |
| **Outer perilesional layer** | f_is_ | 0.55 (0.039) | 0.56 (0.030) | 0.60 (0.015) | **0.005** |
|  | f_in_ | 0.18 (0.030) | 0.16 (0.022) | 0.16 (0.006) | 0.403 |
|  | f_ec_ | 0.27 (0.035) | 0.27 (0.026) | 0.24 (0.018) | **0.009** |
|  | R_s_ | 10.07 (0.149) | 10.03 (0.157) | 10.02 (0.177) | 0.950 |
| **Inner perilesional layer** | f_is_ | 0.55 (0.049) | 0.57 (0.028) | 0.58 (0.017) | 0.469 |
|  | f_in_ | 0.19 (0.030) | 0.17 (0.015) | 0.16 (0.014) | 0.338 |
|  | f_ec_ | 0.27 (0.042) | 0.26 (0.021) | 0.26 (0.019) | 0.880 |
|  | R_s_ | 10.19 (0.140) | 10.18 (0.120) | 10.22 (0.149) | 0.542 |
| **Cortical lesions** | f_is_ | 0.49 (0.089) | 0.48 (0.053) | 0.51 (0.041) | 0.178 |
|  | f_in_ | 0.18 (0.041) | 0.17 (0.031) | 0.15 (0.027) | 0.194 |
|  | f_ec_ | 0.32 (0.086) | 0.35 (0.071) | 0.34 (0.047) | 0.653 |
|  | R_s_ | 10.38 (0.209) | 10.50 (0.131) | 10.43 (0.089) | 0.222 |

Diffusion data were compared within a subset of people with multiple sclerosis (MS) by means of independent samples t-tests. Significant *P*-values (*P*<0.05) are marked in bold.

**Supplementary Table 2** **Results of linear regression models predicting *Z*-scores of average cognition adjusting for age and sex.**

|  | **Average cognition, *Z*-score** |
| --- | --- |
| **Norm. cortical volume, *Z*-score** | 0.16(-0.03;0.35), *P*=0.096 |
| **Cortical lesion volume, log(x)** | -0.07(-0.27;0.14), *P*=0.510 |
| **Cortical SANDI measure, *Z*-score** |  |
| f_is_  f_in_  f_ec_  R_s_ | 0.17(-0.03;0.37), *P*=0.098 |
|  | -0.12(-0.34;0.11), *P*=0.309 |
|  | -0.08(-0.23;0.08), *P*=0.336 |
|  | 0.08(-0.05;0.20), *P*=0.220 |

Separate multivariable linear models were performed with the inclusion of Z-scores of normalized cortical volume, log-transformed cortical lesion volume, and *Z*-scores of intra-soma (f_is_), intra-neurite (f_in_) and extra-cellular (f_ec_) signal fractions and apparent soma radius (R_s_). Beta-coefficients with 95% confidence intervals and corresponding *P*-values are reported.
